# Knowledge of School Nurses on the Basic Principles of Type 1 Diabetes Mellitus Self-Control and Treatment in Children

**DOI:** 10.3390/ijerph192416576

**Published:** 2022-12-09

**Authors:** Anna Stefanowicz-Bielska, Magdalena Słomion, Małgorzata Rąpała

**Affiliations:** 1 Division of Internal and Pediatric Nursing, Institute of Nursing and Midwifery, Faculty of Health Sciences with the Institute of Maritime and Tropical Medicine, Medical University of Gdansk, 80-211 Gdansk, Poland; 2 Department of Pediatric Surgery, Marciniak Hospital, 50-041 Wroclaw, Poland

**Keywords:** diabetes mellitus type 1, child, students, nurses, schools, knowledge

## Abstract

School nurses should participate in the care of children with type 1 diabetes mellitus. The aim of this study was to assess the level of knowledge of school nurses about the basic principles of self-control and the treatment of type 1 diabetes mellitus and to attempt to determine the factors that influence this level of knowledge. A cross-sectional survey was conducted among school nurses from October 2018 to November 2019 in the Pomeranian Voivodeship. The study was conducted using a self-constructed questionnaire. The survey included questions about the sociodemographic characteristics of the respondents, and a test of the knowledge and skills regarding the principles of self-control and the treatment of type 1 diabetes mellitus (16 test questions). This study included 168 Polish school nurses (mean age ± SD = 55.1 ± 8.9 years). Most of the nurses had a secondary education (81%), worked in a municipal educational institution (78.6%), and provided care to more than one student with type 1 diabetes mellitus at school (70.2%). The average level of knowledge of school nurses was 12.5 ± 2.0 points (maximum 16). The nurses working in a village school and those who worked only in one school had lower levels of knowledge. Only 85.7% of nurses reported that they could independently perform a blood glucose measurement with a glucometer, and as many as 56.5% were unable to determine the level of ketone bodies in the urine with Keto-Diastix test strips. Only 62.5% of nurses had a glucometer and glucometer strips in their nursing office. A total of 19.6% of nurses did not have glucagon (1 mg GlucaGen HypoKit^®^, Novo Nordisk A/S, Bagsværd, Denmark) or an ampoule with 20% glucose for an intravenous administration. The knowledge of school nurses about the principles of self-control and the treatment of type 1 diabetes mellitus is insufficient. Due to the strong increase in the incidence of type 1 diabetes mellitus among children and adolescents, it is important to organize permanent, continuous, and mandatory training on the principles of self-control and the treatment of type 1 diabetes mellitus for school nurses. The equipment in Polish school nurses’ offices should be supplemented with a working glucometer and blood glucose test strips, and the set of obligatory medications in the school nurse’s office should be supplemented with glucagon for students with type 1 diabetes mellitus.

## 1. Introduction

Type 1 diabetes mellitus, T1DM, is the most common chronic childhood disease [1]. In Poland, as well as around the world, there is an increase in the incidence of T1DM. It is estimated that approximately 1.2 million children and adolescents aged 0–19 worldwide have the disease, and the number of newly diagnosed cases among youth ages 0–19 is increasing annually by 184,100 [2].

In the last decade, the greatest increase in the incidence of T1DM occurred in Polish children under the age of 15, especially those under the age of 5. The highest increases in the incidence are found in children aged 0–4 years [3].

At diagnosis, children with T1DM often show symptoms of polyuria, polydipsia, and weight loss. Between 10% and 70% of these diagnosed children present in diabetic ketoacidosis [3,4].

The goal of treating diabetes in children and adolescents is to achieve and maintain proper, harmonious physical development, as well as the proper course of adolescence, appropriate to their age and gender, while ensuring a comfortable life for the child and his or her family [4,5].

Insulin therapy continues to be the primary treatment method. Insulin is given using an autoinjector pen or a personal insulin pump [6]. The method of insulin therapy should be adjusted to the individual needs of the child and approved by the patient and their caregivers [5]. A correct dose of insulin depends on the level of glycaemia in the capillary blood or in the interstitial fluid, the dose of carbohydrates consumed, planned physical activity, and the general condition of the patient (the additional factors affecting the dose of insulin are an illness or stress) [5]. Despite the implementation of modern methods of self-control and therapy, children with T1DM are still at risk of acute metabolic disorders, such as mild, moderate, and severe hypoglycemia, as well as hyperglycemia and ketoacidosis. In children and adolescents with T1DM, the recommended target HbA1c is ≤6.5% with stable blood glucose levels, the minimization of hypoglycemic episodes and the maintenance of a good quality of life; time spent in the target glycaemia (TIR—time in range) should be >80%. Achieving treatment goals from the very beginning of the disease may prevent the occurrence of acute and chronic complications and enable a normal and active family, professional, and social life [5].

Studies of the Diabetes Control and Complications Trial Research Group and the Epidemiology of Diabetes Interventions and Complications Research Group have shown that achieving and maintaining glucose levels leads to a reduction in the micro- and macro-vascular complications in T1DM [7,8].

To achieve optimal treatment results, children with diabetes should be cared for by a multidisciplinary team that includes diabetes educators, diabetes nurses, nutritionists, physiotherapists, social workers, psychologists, diabetologists, and other physicians (e.g., pediatricians, general practitioners, and emergency physicians). The cooperation of the diabetic therapeutic team with the school nurse and the family is aimed at providing a child with T1DM with the best care and the greatest possible safety in an educational institution.

The aim of the study was to assess the level of knowledge of school nurses about the disease, the basic principles of treatment, and the occurrence of acute metabolic disorders and complications, as well as to determine the factors influencing their level of knowledge.

## 2. Materials and Methods

### 2.1. Study Design, Setting and Participants

This study was a cross-sectional survey conducted from 23 October 2018 to 30 November 2019 in the Pomeranian Voivodeship. The study participants were 168 professionally active school nurses. The data were collected during individual meetings with the school nurses or at a training conference organized by the Regional Council of nurses and midwives for school nurses. The inclusion criteria were active school nurses working in school, and the exclusion criteria were all nurses who do not work in schools.

### 2.2. Methods

The study was conducted using a diagnostic survey method with a self-constructed questionnaire. When preparing the questionnaire, the factors that may affect the level of knowledge of school nurses were considered.

The first page of the questionnaire contained information about the study and an invitation to participate. The survey questionnaire was divided into four parts.

The first part of the survey contained questions about the respondent (the surveyed school nurse), i.e., sociodemographic data such as their age, education, whether they had completed postgraduate education (specialist training in pediatric nursing and family nursing and qualification courses in pediatric nursing, diabetes nursing, family nursing, diabetes educator, care for people with diabetes using continuous subcutaneous insulin infusion (CSII), and caring for children and adolescents with diabetes), whether they had completed postgraduate education in the field of nursing in an educational setting (specialization training in nursing in an educational setting, the qualification course in nursing in an educational setting), their workplace, and the number of facilities where the nurse works.

The second part of the questionnaire consisted of questions about the characteristics of the school where the nurse works: the presence of a student with T1DM in school, the number of students with T1DM in school, the method of diabetes treatment used, the presence of an additional diseases in a student with T1DM, telephone contact with the parents of a sick child, and the equipment of the nursing office.

The third part of the questionnaire included questions about the nurses’ skills and preparation for caring for a child with T1DM and whether they would like to participate in training for the control and treatment methods of type 1 diabetes, such as: can you independently measure the blood glucose level with a blood glucose meter? Can you measure the level of ketone bodies in the urine with test strips? What will you do if a student with T1DM becomes unconscious? How do you rate your preparation for caring for a student with T1DM? Would you like to repeat the training on the causes, symptoms, acute metabolic disorders, complications, and treatment methods for T1DM? What kind of training do you think would be the best for you?

The fourth part of the questionnaire included a theoretical test on type 1 diabetes.

The knowledge test consisted of 16 test questions concerning the essence of the disease (the definition of diabetes and the essence of type 1 diabetes), the basic principles of self-control and treatment (how insulin works, nutrition of children with T1DM, the percentage of different nutrients in the meal, the main source of carbohydrates, the definition of the carbohydrate unit, the types of carbohydrates, physical exercise as an integral part of diabetes treatment, the regularity of physical exertion, rules applicable at the time of undertaking physical exercise, and the recommended type of exercise) and the occurrence of acute metabolic disorders and complications (the management of severe hypoglycemia, acute metabolic disorders in T1DM, indications for glucagon administration, and the symptoms of hyperglycemia).

The test to check the level of knowledge was constructed based on the recommendations of the Polish Federation for Education in Diabetes and the Polish Diabetes Association. Each question contained one correct answer, for which the nurses received one point. A maximum of 16 points could be obtained.

### 2.3. Data Collection

The data were collected using a paper version of the questionnaire. The questionnaires were distributed and collected personally by the researcher during individual meetings with school nurses or at a training conference organized by the Regional Council of nurses and midwives for school nurses. Participation in the study was voluntary.

### 2.4. Statistical Analysis

For each parameter, the mean (X), median (M), standard deviation (SD, range (min, max), and lower and upper quartile (25Q, 75Q) were calculated. The statistical significance between the means for different groups was calculated by a one-way analysis of variance (ANOVA), alternatively using the non-parametrical U Mann–Whitney test (for two groups) or Kruskal–Wallis test (for more than two groups), when the variances in the groups were not homogeneous (the homogeneity of variance was determined by the Levene’s test.

The relation between the two parameters (the level of knowledge versus the age) was assessed using a correlation analysis and Spearman correlation coefficients were calculated.

A *p* value of less than 0.05 was required to reject the null hypothesis. Statistical analysis was performed using EPIINFO Ver. 7.2.3.1 and Statistica Ver. 13.3. software packages.

## 3. Results

### 3.1. Characteristics of the Study Participants

The study included 168 Polish school nurses from the Pomeranian Voivodeship (2,346,717 inhabitants, 776 primary schools, 84 level I vocational schools, 143 general secondary schools, 106 technical secondary schools, 12 art schools, and 27 vocational schools for students with disabilities) [9,10].

Table 1 presents the characteristics of the study participants.

### 3.2. The Level of Knowledge about the Essence of the Disease, the Basic Principles of Self-Control and Treatment and Acute Metabolic Disorders and Complications

The average result of the test checking the level of knowledge of the school nurses was 12.5 ± 2.0 points (maximum 16).

The results of the knowledge test are presented in Table 2.

Questions 4 and 5 were the most difficult. Question 4 concerned the basic principles of rational nutrition, and question 5 concerned the percentage composition of particular nutrients in the proper diet of a child with T1DM. The correct answer was given by only 25% and 58.3% of the respondents, respectively.

The highest number of correct answers was obtained for questions 3 (94.6%), 6 (96.4%), 9 (96.4%), 13 (97.6%), and 14 (92.3%). Question 3 related to the definition of type 1 diabetes, question 6 related to the knowledge of the main sources of carbohydrates, question 9 assessed the general knowledge of exercise as an integral part of the proper management of T1DM, question 13 related to the type of exercise recommended, and question 14 checked the knowledge of acute metabolic disorders in T1DM that threaten the life of a student with T1DM.

### 3.3. Nurses’ Self-Assessment of Skills and Preparation for Caring for a Child with Type 1 Diabetes

Most nurses (144/168, 85.7%) reported that they could independently perform blood glucose measurements with a glucose meter. However, 95/168 (56.5%) could not measure urine ketones with Keto-Diastix test strips. Most nurses (95.2%, 160/168) knew what to do if a student with type 1 diabetes became unconscious.

A slight majority of the nurses (62.5%, 105/168) had a blood glucose meter and blood glucose meter strips in their offices. Approximately 19.6% (33/168) of nurses did not have glucagon (GlucaGen 1 mg HypoKit^®^) or an ampoule with 20% glucose for an intravenous administration in their offices. A total of 16.7% (28/168) of nurses did not have a telephone number to contact the parents of a student with T1DM.

A total of 13.1% (22/168) of nurses assessed their preparation for caring for a student with T1DM as low, 75.6% (127/168) as average, and 11.3% (19/168) as high.

The majority of nurses (70.2%, 118/168) said they would like to repeat the training on the causes, symptoms, methods of preventing acute metabolic disorders, methods of self-control, and the treatment of type 1 diabetes. Only 25% of nurses (42/168) would like it to receive individual training.

### 3.4. Analysis of Factors That May Affect the Level of Knowledge

The authors analyzed the influence of various factors on the level of knowledge of school nurses.

Nurses who were employed in only one school and those who worked in a rural school had a lower level of knowledge (Table 3).

## 4. Discussion

School and preschool activity is a very important part of the life of every child and young adult with type 1 diabetes [11,12,13]. The educational institution recognizes the position of the child in the family and environment, stimulates the individual and social development of the child, and integrates and teaches group life. A child with T1DM should attend school or kindergarten regularly [10]. This approach poses challenges for the patient’s caregivers and peers.

Close cooperation between children, parents, school staff, and health care providers is needed to ensure that children and adolescents with T1DM are motivated to participate actively in all educational activities [14].

The results of the largest clinical trial, the Diabetes Control and Complications Trial (DCCT), clearly demonstrated that the intensive therapy of T1DM to reduce blood glucose to the normal range is associated with a significant reduction in the risk of developing microvascular complications, retinopathy, and neuropathy [7].

Based on these studies, the American Diabetes Association concluded that intensive care should be a method of treatment for all children with T1DM over the age of 13. The intensive treatment of type 1 diabetes improves the quality of life of children and adolescents with this disease.

The above recommendations significantly changed the role of the school nurse in caring for children with T1DM in educational institutions.

The school nurse has become one of the key elements determining the safety of a child with type 1 diabetes at school.

School nurses should be responsible for the organization and implementation of medical care for children with T1DM in school or kindergarten. They are partners for children and adolescents with T1DM and their parents in the implementation of the treatment and self-monitoring process [15,16].

In schools, the proper organization of diabetes care requires the training of all school staff on type 1 diabetes and the principles of its treatment, including the acceptance of the principles of self-control and joint responsibility for the organization of diabetes care [15,17,18].

The aim of our study was to assess the level of knowledge of school nurses on the essence of the disease, the basic principles of treatment, and the occurrence of acute metabolic disorders and complications. It was also an attempt to determine the factors influencing their level of knowledge. The research was carried out with 168 school nurses. The average age of the respondents was 55.1 ± 8.9 years. Most nurses had secondary education (81%), worked in a municipal educational institution (78.6%), and cared for more than one student with T1DM (70.2%). As many as 13% of nurses did not know what method a student with T1DM was treated with, and approximately 17% did not have a phone number to contact the parents of a child with T1DM.

Only 85.7% of nurses were able to perform a blood glucose test with a glucose meter, but 56.5% were unable to determine the level of ketone bodies in the urine with the Keto-Diastix test strips.

More than half of the nurses (62.5%) had a blood glucose meter and blood glucose meter strips in their offices. A total of 19.6% of nurses did not have glucagon (GlucaGen 1 mg HypoKit^®^) or a 20% glucose ampoule for an intravenous administration in their offices.

In our study, the state of knowledge was analyzed on the basis of the test, which included 16 test questions concerning the essence of the disease, the basic principles of self-control and treatment, and acute metabolic disorders and chronic complications. Each question contained one correct answer, for which the nurses received one point. A maximum of 16 points could be obtained.

The average level of knowledge of school nurses was 12.5 ± 2.0 points. The most difficult questions were questions about the basic principles of rational nutrition (25% correct answers) and the percentage of particular nutrients in a meal for a child with T1DM (58.3% correct answers).

In the literature, seven studies were found on the assessment of the level of knowledge, competence, and self-efficacy in the field of diabetes therapy in school nurses.

Surveys concerning the assessment of the level of knowledge of school nurses on the self-control and treatment of T1DM were conducted in Poland, Kuwait and the United States.

The Polish study by Kobos et al. presents the actual and perceived level of knowledge about diabetes among 230 Warsaw school nurses. It concerns school nurses working in schools of one of the largest cities in Poland (Warsaw with a population of 1,794,200). The percentage of correct responses in the Diabetes Knowledge Questionnaire was 46.7%, with the lowest scores related to the knowledge of insulin pumps (36.5%), nutritional principles (37.4%), and insulin therapy and glucagon administration (37.9%). The actual and perceived diabetes knowledge were moderately positively correlated (rho = 0.18; *p* = 0.009). School nurses perceived their knowledge of diabetes to be better than their actual knowledge. Scores on the Diabetes Awareness Questionnaire were higher in nurses with a higher education (*p* = 0.024), those who had relatives or friends with diabetes (*p* = 0.032), and those who had previously received diabetes training (*p* = 0.05) [19,20].

In our study, not all nurses were able to independently perform blood glucose measurements with a glucometer and determine the level of ketone bodies in the urine with Keto-Diastix test strips, and not all knew what to do if a student with T1DM lost consciousness. Only 11.3% of nurses rated their preparation for caring for a student with T1DM as high. It was shown that nurses who were employed in only one school and those who worked in a rural school had a lower level of knowledge.

Research conducted in Kuwait and the United States shows the impact of educational training on the level of knowledge and skills in the principles of self-control and treatment of T1DM among nurses and school workers.

Tahaa et al. assessed the impact of e-learning training on the level of knowledge of school workers in Kuwait. A total of 124 employees (52–55.3% teachers, 14–14.9% school nurses, 18–19.2% administrative employees, and 10–10.6% psychologists) from 31 public schools participated in the training. Before and after the diabetes training, the participants were asked to complete a questionnaire verifying their level of knowledge about diabetes. The authors of the study stated that the online training significantly increased the level of knowledge of the school staff about diabetes. The level of diabetes awareness remained the same 12 months after completing the online training. The school staff showed significantly greater self-confidence while caring for students with diabetes [21].

In Colorado, the United States, Berget et al. assessed the diabetes care provided by schools and the necessary knowledge and skills of school medical personnel and explained the role of school medical personnel in diabetes care. The level of knowledge, competence, and self-efficacy in the field of diabetes therapy was assessed before and after the course. The study included 30 school nurses. The level of knowledge, competence, and self-efficacy in the treatment of diabetes improved significantly after the implementation of the diabetic course. The level of diabetes knowledge increased by 3.5% (*p* = 0.01), and the sense of self-efficacy improved by 2.7% (*p* = 0.007). Researchers found that developing the competencies of school medical personnel through intensive training and the practical use of specialist knowledge can help organize and provide the best diabetes care in an educational institution [22].

Other American researchers tried to evaluate the effectiveness of the educational program Helping Administer to the Needs of the Student with Diabetes in Schools (H.A.N.D.S.^SM^) implemented in Chandler, Arizona, Columbus, Ohio, El Paso, Texas, Kingston, New York, Naperville, Illinois, Queensbury, San Diego, and California. H.A.N.D.S.^SM^ is a continuing education program aimed at improving the level of experience and competence in performing the services associated with diabetes care. The level of experience and competence in the field of diabetes care was assessed among 105 school nurses before and after the training. The survey was conducted via email. The participants’ levels of experience and competence for each of the four categories of diabetes care improved significantly, and a greater number of nurses reported being able to perform the services independently and having the ability to teach others [23].

Rhodes et al. assessed the effectiveness of e-learning training for school nurses on the latest guidelines for school diabetes care developed by the National Diabetes Education Program (2016 NIDDK). A total of 1977 school nurses in Missouri were invited to participate in the training, and the researchers assessed their initial and final levels of knowledge. A total of 678 school nurses completed the entrance test, and 449 completed the final test. The authors of the study found that the level of knowledge of school nurses increased significantly compared to the initial level. The training provided personalized, flexible, computerized information on diabetes and care plans. In their conclusions, the authors emphasized that e-learning is a convenient and effective way of providing professional development opportunities and raising the knowledge of school nurses [24].

In Normandy, an online continuing education program for school nurses on self-management and diabetes management for students was implemented and evaluated using current policies. Nineteen school nurses who were unable to attend on-site diabetes training across the state were recruited for the study. Most of the school nurses stated that the goals had been achieved. A total of 91% of respondents said that the education would improve their skills of coping with students who had diabetes. The authors of the study emphasized that due to the increasing prevalence of diabetes in children, it is important that school nurses have constant access to lifelong learning, which includes the current codes of practice in children with diabetes [25].

In Indiana, the United States, a survey was conducted among school workers to assess the effectiveness of a basic and extended education program on the principles of therapy and the self-management of T1DM. This project examined the initial and final knowledge of school workers about the principles of therapy and the self-management of T1DM and their confidence in caring for students with diabetes. Forty-four employees participated in the basic training, and 37 participated in the extended training, including 22 (59.5%) aides/assistants, 5 (13.5%) clerks, 3 (8.1%) nurses, and 7 (18.9%) other staff. The authors of the study showed that the general knowledge about the self-control and treatment of diabetes and the self-confidence in caring for a student with diabetes increased significantly after both basic and extended training compared to the initial level [26].

It is very important to underline that on an ongoing basis, school nurses identify the health problems of students with T1DM, formulate nursing diagnoses, and conduct nursing assessments; they develop, implement, coordinate care plans for individuals, groups, parents, and teachers, and assess the necessary supplies for medical care services [27,28].

### Study Limitations

The main limitations of this study were as follows: the questionnaire was not tested for its validity and reliability, and the study was performed only in one region of Poland.

## 5. Conclusions

The knowledge of school nurses about the principles of self-control and the treatment of type 1 diabetes is insufficient. Due to the intense increase in the incidence of type 1 diabetes in children and adolescents, it is important to organize permanent, continuous, mandatory training on the principles of self-control and the treatment of type 1 diabetes for school nurses. Politicians, directors of medical units, and school principals should ensure a safe school environment for students with type 1 diabetes and implement appropriate interventions to support them, including methods for resolving the health, psychological, and social problems. It is necessary to provide all students with type 1 diabetes with access to medical services provided by nurses. The equipment of the Polish school nurse’s office should be supplemented with a working glucometer and blood glucose test strips, and in the case of the presence of a child with type 1 diabetes among students at school, a set of obligatory medications from the school nurse’s office should be supplemented with glucagon.

## Figures and Tables

**Table 1:** Characteristics of school nurses.

Demographic Data		Mean (X) ± SD/N (%)
Age [years]		55.1 ± 8.9
Place of work	Village	36 (21.4%)
	City	132 (78.6%)
Number of facilities where the school nurse works	One	113 (67.3%)
	More than one	55 (32.7%)
Education	Nursing secondary school	136 (81%)
	Bachelor of nursing	20 (11.9%)
	Master of nursing	12 (7.1%)
Completed postgraduate education	Yes	138 (82.1%)
	No	30 (17.9%)
Completed postgraduate education in the field of nursing in an educational setting	Yes	125 (74.4%)
	No	43 (25.6%)
The presence of a student with T1DM ^a^ in school	Yes	146 (86.9%)
	No	22 (13.1%)
Number of students with T1DM ^a^ in school	None	22 (13.1%)
	One	28 (16.7%)
	More than one	118 (70.2%)
The presence of an additional chronic disease in a student with T1DM ^a^	No answer	22 (13.1%)
	Yes	51 (30.4%)
	No	95 (56.5%)
Method of treating a student with T1DM ^a^	No answer	22 (13.1%)
	Personal insulin pump	69 (41.1%)
	An autoinjector pen	38 (22.6%)
	Both methods	39 (23.2%)

^a^ type 1 diabetes mellitus.

**Table 2:** Assessment of the knowledge of school nurses on the basis of a knowledge test (number and % of correct and incorrect answers).

Questions	% of Answers			
	Correct		Incorrect	
	N	%	N	%
Definition of diabetes	145	86.3%	23	13.7%
How insulin works	148	88.1%	20	11.9%
Essence of type 1 diabetes	159	94.6%	9	5.4%
Nutrition of children with T1DM ^a^	42	25%	126	75%
Percentage of different nutrients in the meal	98	58.3%	70	41.7%
Main source of carbohydrates	162	96.4%	6	3.6%
Definition of the carbohydrate unit	143	85.1%	25	14.9%
Types of carbohydrates	154	91.7%	14	8.3%
Physical exercise as an integral part of diabetes treatment	162	96.4%	6	3.5%
Regularity of physical exertion	150	89.3%	18	10.7%
Rules applicable at the time of undertaking physical exercise	131	78%	37	22%
Management of severe hypoglycemia	146	86.9%	22	13.1%
Recommended type of exercise	164	97.6%	4	2.4%
Acute metabolic disorders in T1DM ^a^	155	92.3%	13	7.7%
Indications for glucagon administration	146	86.9%	22	13.1%
Symptoms of hyperglycemia	143	85.1%	25	14.9%

^a^ type 1 diabetes mellitus.

**Table 3:** Influence of various factors on the level of knowledge of school nurses.

Influencing Factor		N	Level of Knowledge	*p* Test
Age		168	−0.05	0.492 ^a^
Nurse’s place of work	Village	36	12 (10 ÷ 13) **	0.000 ^b^
	City	132	13 (12 ÷ 14) **	
Number of facilities where the school nurse works	One	113	13 (11 ÷ 14) **	0.016 ^b^
	More than one	55	13 (12 ÷ 14) **	
Education	Secondary nursing education	136	13 (12 ÷ 14) **	0.891 ^c^
	Bachelor of nursing	20	13 (10.5 ÷ 14) **	
	Master of nursing	12	13 (12 ÷ 14) **	
Completed postgraduate education in the field of nursing in an educational setting	Yes	125	12.3 ± 2.2 *	0.149 ^d^
	No	43	12.8 ± 1.5 *	
The presence of a student with T1DM ^e^ in school	Yes	146	13 (12 ÷ 14) **	0.115 ^b^
	No	22	11 (9 ÷ 14) **	
Method of treating a student with T1DM ^e^	Personal insulin pump	69	13 (12 ÷ 14) **	0.946 ^b^
	Autoinjector pen	38	13 (12 ÷ 14) **	

* Mean (X) ± SD, ** M (25Q ÷ 75Q), ^a^ R Spearman correlation, ^b^ U Mann–Whitney test, ^c^ Kruskal–Wallis test, ^d^ ANOVA, ^e^ type 1 diabetes mellitus.

## Data Availability

Data available on request due to privacy restriction.
